# Association Between Tremor Severity and Caregiving Intensity in Essential Tremor

**DOI:** 10.5334/tohm.1046

**Published:** 2025-08-07

**Authors:** Margaret E. Gerbasi, Rodger J. Elble, Holly A. Shill, Eddie Jones, Alexander Gillespie, John Jarvis, Elizabeth Chertavian, Zachary Smith, Ludy C. Shih

**Affiliations:** 1 Sage Therapeutics Inc., Cambridge, MA, US; 2 Southern Illinois University School of Medicine, Springfield, IL, US; 3 Barrow Neurological Institute, Phoenix, AZ, US; 4 Adelphi Real World, Adelphi Mill, Bollington, UK; 5 Medicus Economics, Milton, MA, US; 6 Beth Israel Deaconess Medical Center, Boston, MA, US; 7 Harvard Medical School, Boston, MA, US

**Keywords:** essential tremor, caregiving intensity, activities of daily living

## Abstract

**Background:**

Essential tremor (ET) is a common movement disorder that affects the upper limbs in vital activities of daily living. Few studies have examined the impact of tremor severity on caregiving intensity.

**Methods:**

Clinic-based data were collected in the United States (US) from March 2021 to August 2021 through the Adelphi ET Disease Specific Programme (DSP). Linked data between physician and care partner/patient pairs were used to evaluate care partner-reported weekly hours of patient care. Tremor severity was assessed with the Essential Tremor Rating Assessment Scale (TETRAS) Performance and Activities of Daily Living subscales. Pearson chi-square, correlation and multivariate regression analyses were conducted to examine the relationships between tremor severity and hours of care partner assistance.

**Results:**

A quarter of 960 individuals with ET required care partners. Most care partners were spouses (61%), but other family members or friends also served as care partners. About 23% of care partners reported giving constant care, defined as 112 hours per week or more, while the remainder of care partners reported caregiving time averaging 24.5 hours per week. The probability of needing a care partner was significantly associated with tremor severity, and the association between care partner need and tremor severity was moderate (bivariate r = 0.32–0.37) and not substantially impacted by the inclusion of additional covariates (age, sex, race, comorbidity, relationship with patient, and living with patient).

**Discussion:**

Roughly 25% of patients with ET have care partner needs, and the number of care partner hours provided is correlated with tremor severity. Therefore, treatments that ameliorate tremor severity have the potential to reduce caregiving intensity in ET.

## Introduction

Essential tremor (ET) is among the most common movement disorders in the US, affecting an estimated 6.8 million US adults [1]. Primary manifestations of ET include kinetic and postural tremor of the upper limbs [2]. Tremor severity is progressive [3], with increasing frailty [4] and decreasing physical activity [5] seen in individuals with ET. People with ET often struggle to perform activities of daily living (ADL) [6,7,8,9], and it is understandable that they may rely on care partners for physical and emotional support. While ET burden on the healthcare system is becoming increasingly recognized [8,10,11], the factors driving the indirect care needs of patients with ET have been studied only in few patient cohorts. Several studies have assessed care partner burden in ET, as well as its predictors, including both motor and non-motor symptoms [12,13,14]. One study found a correlation between care partner burden and personal embarrassment from ET [12], and also between care partner burden and non-motor symptoms, such as decreased cognitive function and increased depressive symptoms [13], but no correlation was found between care partner burden and tremor severity [12,13].

The lack of association between care partner burden and tremor severity is counterintuitive and was observed in a small study sample (N = 57) of adults age ≥ 55 who consented to participate in a longitudinal study of cognitive function and eventual brain donation [12,13]. However, while care partner burden has been defined as “a series of negative responses that occur while undertaking the role of primary caregiver [15],” caregiving intensity is defined in terms of time costs, “the amount of informal care provided every day or per week and is often used as a key predictor for working age caregivers’ labor market penalties [16].” To our knowledge, no other studies have directly assessed the need for care partner and caregiving intensity, expressed as the number of hours reported as being provided by a care partner among a clinical population of ET patients and their care partners. Therefore, we examined the relationship between caregiving intensity (i.e., number of hours spent) and tremor severity in a large observational clinical dataset involving physicians, care partners, and adult patients of any age.

## Methods

**Figure 1:**
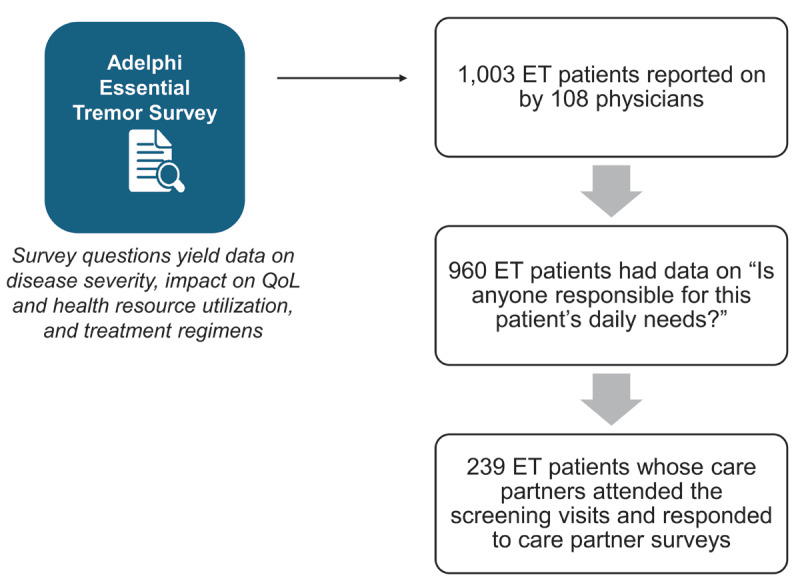
**Adelphi ET Disease Specific Programme (DSP) analytic sample**. Flow diagram for the ET DSP sample. Beginning with 1,003 ET patients on whom data was available, 960 ET patients had complete information on whether caregiving was needed, and 239 ET patients had a unique care partner available to answer care partner surveys at the study visit.

This study used real-world data collected in the United States (US) through the Adelphi ET Disease Specific Programme (DSP) [17]. The Adelphi Disease Specific Programme consists of large, multi-national, observational studies that are designed to investigate real-world observational clinical data from participating physicians and patients. Data were collected in the US from March 2021 to August 2021, resulting in de-identified data from 1,003 ET patients and 98 treating physicians comprised of 38 primary care physicians and 60 neurologists (Figure 1). Physicians involved in the management of patients with ET in the US were identified from public lists of healthcare professionals and included in the study if they were current practicing physicians who treated 10 or more patients with diagnosed ET in a typical month. Each eligible and consenting physician provided information on their next 10 consecutive patients with ET, regardless of the reason for the visit. Patients with ET were ≥18 years old and not currently involved in a clinical trial.

Need for care was assessed by the question “Is anyone responsible for this patient’s daily needs?” which was completed by the physicians on 960 patients. The answer was affirmative in 240 of these 960 patients. Separately, for 253 patients, a unique care partner was identified by the patient, and 239 of these care partners completed a care partner survey, including reporting the weekly hours spent in patient care. These patient/care partner dyads comprised a subset sample, “Patient/Care Partner cohort.” The weekly hours spent on patient care was assessed with the question “How long, on average, do you care for this patient for their Essential Tremor?”. Care partners were given the option to provide the open-ended number of hours per day, open-ended number of hours per week, or to select “Constantly”. To analyze time needed for caregiving on a continuum, if constant care was reported, time commitment was assumed as 112 hours/week (e.g., 2/3 of total weekly hours, or 16 hours/day for 7 days total). Daily hours were multiplied by 7 to yield an estimate of hours/week. Care partner patient care was categorized as high-intensity if the hours per week exceeded 20 based upon prior work in several foundational studies of caregiver burden defining high-intensity caregiving as 20 hour or more per week [18,19,20,21]. Care partners were also surveyed to assess the need for additional resources, home modifications, and additional assistance.

Tremor severity was assessed with the Essential Tremor Rating Assessment Scale (TETRAS) Activities of Daily Living (ADL) and Performance (P) subscales [22]. TETRAS-P quantifies action tremor of the head, face, voice, upper limbs and lower limbs with 0-4 point ratings, but 56 of the maximum possible 68 points are derived from upper limb assessments. TETRAS-P item 4 measures right and left-hand tremor in the forward horizontal posture, wing posture, and finger-nose-finger task and has a maximum score of 24 points [22]. In TETRAS-ADL, a clinician interviews the patient regarding the impact of tremor on speech (one item), upper limb function (10 items), and social function (one item) and scores each item 0–4 [22]. Thus, 40 of the maximum 48 points are attributable to upper limb tremor.

Pearson correlations and multivariate regression models, adjusting for relevant covariates (TETRAS subscale of interest, age, sex, race, Charlson Comorbidity Index [CCI] [23]) were used to assess associations between care provision and tremor severity as measured by TETRAS-P, TETRAS-P item 4, and TETRAS-ADL. For analyses modeling the need for a care partner, bivariate regression included only the TETRAS variable of interest as the predictor variable. The core multivariate logistic regression model covariates additionally included age, sex, race, and CCI, and the expanded model covariates also included core model covariates plus employment status. For analyses on time spent caregiving, bivariate regression included only the TETRAS variables of interest as predictor variables. Core model covariates additionally included age, sex, care partner’s relationship to patient, and whether the care partner lived with the patient. Expanded model covariates also included core model covariates plus care partner age and care partner employment status.

## Results

Of the 1,003 patients included in the ET DSP, 960 patients had care partner status reported and were included in the main analysis dataset. The ET patients were comprised of 453 females (47%), and the mean age of the overall cohort was 64.9 (standard deviation, SD, 13.3) years old, with 725 patients reported to be White (75.5%) 111 African American (11.6%), 47 Asian (4.9%), and 35 Hispanic (3.6%) (Table 1).

**Table 1:** Demographics and ET disease characteristics.


		OVERALL ET PATIENTS (n = 960)		PATIENT/CARE PARTNER COHORT (n = 239)	

Sex, % (n)	Female	47%	(453)	49%	(116)

Age (years), mean (sd)	Age	64.9	(13.3)	67.9	(11.6)

BMI, mean (sd)	BMI	26.6	(4.6)	26.0	(4.0)

Age category, % (n)	18-64	43.1%	(414)	35.6%	(85)

	64+	56.9%	(546)	64.4%	(154)

Race/Ethnicity, % (n)	White/Caucasian	75.5%	(725)	74.1%	(177)

	Asian	4.9%	(47)	5.9%	(14)

	African American	11.6%	(111)	8.8%	(21)

	Hispanic	3.6%	(35)	4.6%	(11)

	Other	4.4%	(42)	6.7%	(16)

Insurance coverage, % (n)	Medicare	50.4%	(484)	57.7%	(138)

	Medicaid	5.0%	(48)	4.2%	(10)

	Commercial	40.9%	(393)	35.1%	(84)

	Other	3.6%	(35)	2.9%	(7)

Employment status, % (n)	Full time	31.9%	(306)	23.8%	(57)

	Part time	9.4%	(90)	8.8%	(21)

	Retired	46.3%	(444)	50.2%	(120)

	Unemployed	11.8%	(113)	17.2%	(41)

	Student	0.7%	(7)	0.0%	(0)

Current home circumstances, % (n)	Residing at a nursing home	1.5%	(14)	1.7%	(4)

	Residing with family	82.9%	(796)	89.1%	(213)

	Residing alone	14.5%	(139)	7.1%	(17)

	Other	0.5%	(5)	1.7%	(4)

	Unknown	0.6%	(6)	0.4%	(1)

Has someone responsible for daily needs, % (n)	Yes	25.0%	(240)	65.7%	(157)

	No	75.0%	(720)	30.5%	(73)

	Unknown	0.0%	(0)	3.8%	(9)

TETRAS scores, mean (sd)	TETRAS activities of daily living (ADL) score	16.2	(9.4)	18.8	(9.8)

	TETRAS Performance score	21.4	(11.7)	24.3	(12.1)

	TETRAS Performance Item 4 score	9.4	(4.7)	10.6	(4.8)

	TETRAS total score	37.6	(20.4)	43.2	(20.9)

Onset of ET symptoms, % (n)	Childhood	2.2%	(21)	0.8%	(2)

	20–29	4.0%	(38)	2.1%	(5)

	30–39	5.4%	(52)	2.1%	(5)

	40–49	12.3%	(118)	9.6%	(23)

	50–59	24.4%	(234)	28.0%	(67)

	60–69	29.9%	(287)	34.7%	(83)

	70–79	11.6%	(111)	13.8%	(3)

	80–89	2.2%	(21)	2.9%	(7)

	Unknown	8.1%	(78)	5.9%	(14)

ET treatment status/history, % (n)	Currently prescribed a drug for treating ET	83.9%	(805)	90.0%	(215)

	Previously prescribed a drug for treating ET	7.3%	(70)	3.3%	(8)

	Has never been prescribed ET treatment	8.9%	(85)	6.7%	(16)

Currently-prescribed ET treatments, % (n)	Propranolol	38.9%	(373)	40.6%	(97)

	Primidone	31.4%	(301)	39.3%	(94)

	Atenolol	7.6%	(73)	10.5%	(25)

	Sotalol	0.2%	(2)	0.8%	(2)

	Nadolol	1.6%	(15)	3.3%	(8)

	Alprazolam	5.4%	(52)	7.9%	(19)

	Clonazepam	7.5%	(72)	8.4%	(20)

	Lorazepam	3.0%	(29)	3.8%	(9)

	Diazepam	2.4%	(23)	4.6%	(11)

	Gabapentin	5.8%	(56)	6.7%	(16)

	Pregabalin	2.5%	(24)	3.8%	(9)

	Topiramate	7.2%	(69)	7.5%	(18)

	Zonisamide	0.7%	(7)	0.0%	(0)

	Clozapine	0.2%	(2)	0.0%	(0)

	Nimodipine	0.0%	(0)	0.0%	(0)

	Botulinum toxin	2.7%	(26)	3.3%	(8)

	Deep-brain stimulation (DBS)	0.5%	(5)	0.8%	(2)

	Cala Trio (wrist-worn device)	2.4%	(23)	3.8%	(9)

	Other	2.0%	(19)	1.3%	(3)

Past ET-related procedures, % (n)	Deep-brain stimulation (DBS)	5.8%	(56)	7.1%	(17)

	Thalamotomy	3.3%	(32)	2.5%	(6)

	Magnetic resonance-guided focused ultrasound (MRgFUS)	4.4%	(42)	3.8%	(9)

	Other	2.6%	(25)	2.5%	(6)

	None	77.9%	(748)	77.8%	(186)

	Unknown	13.1%	(126)	10.9%	(26)


BMI, body mass index; TETRAS, The Essential Tremor Rating and Assessment Scale.

**Figure 2:**
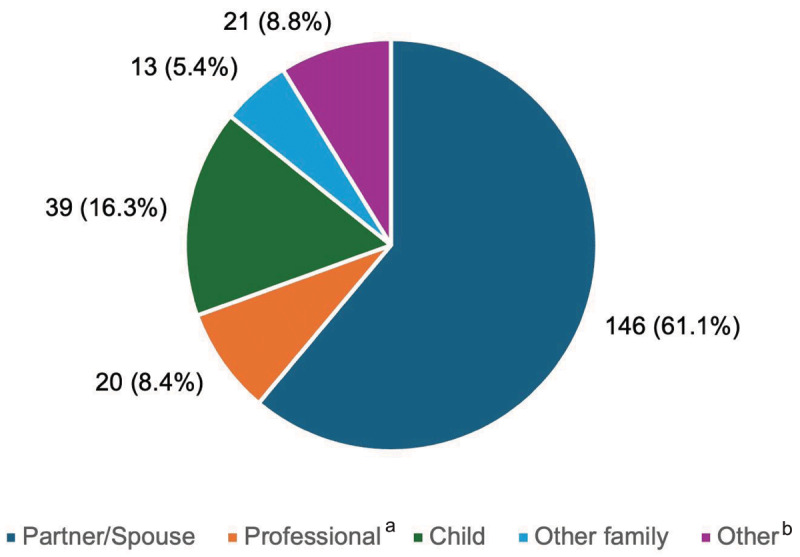
**Relationships of care partners to individuals with ET**. ^a^Professional = nursing home staff, nurse, home help, or professional care partner; ^b^Other = voluntary care partner, friend/neighbor, other.

In this cohort, 25% (240/960) required care partners by physician report. Patients who required care partners were on average older than those without care partners (mean age, 70.8 vs 63.0 years; p < 0.001). Compared with the mean age of ET patients and based upon the survey responses directly from care partners (n = 239), care partners were on average younger, 56.9 years of age (SD, 14.4 years, Supplementary Table). Most care partners were a spouse or domestic partner (146/239; 61.1%) of the patient with ET (Figure 2), followed by a child (39/239; 16.3%) or a voluntary care partner, friend/neighbor, or other person (21/253; 8.8%). Most care partners were employed or students (146/239; 61.1%) and lived with the patient (196/239; 82.0%).

Constant care for patients with ET was provided by 22.6% (54/239) of care partners. Excluding care partners who reported providing constant care (time commitment defined as 112 hours/week), weekly care partner time averaged 24.5 hours (SD, 26.1). Including constant-care care partners resulted in an increase in the mean amount of time devoted to care to 43.9 weekly care hours, with more than half (148/239; 61.9%) of care partners giving at least 20 hours of care per week. Care partners only reported the amount of caregiving time that they themselves provided, but 92% of care partners reported that their patient had at least one additional care partner.

### Association between care partner need and measures of tremor severity

**Figure 3:**
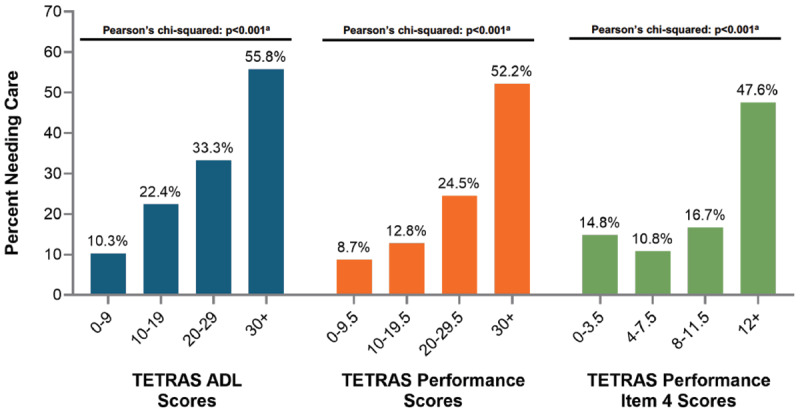
**Association between proportion of individuals with ET with care needs and tremor severity**. ^a^Pearson chi-square testing a global null hypothesis that distributions are equal across the 4 subcategories within a given TETRAS measure. The p-values < 0.05 indicate that the percentage of patients needing care is not equal across the subgroups.

Greater tremor severity was associated with a higher probability of needing a care partner (Figure 3). As TETRAS-P, TETRAS-ADL and TETRAS-P Item 4 scores increased (i.e., worsening tremor), the proportion of patients requiring care partners increased significantly (Pearson’s chi-squared, p < 0.001). The association between the need for a care partner and tremor severity was moderate (bivariate r = 0.32–0.37), and the associations were not substantially impacted by the inclusion of age, sex, race, comorbidity, as covariates in expanded regression models (Table 2). Sensitivity analyses were conducted to include employment status of the ET patient as a covariate in the models, and this did not change the significant association between tremor severity and the need for a care partner. Finally, other factors were significantly associated with the need for care partner, including a higher degree of medical co-morbidity as measured by the Charlson Comorbidity Index.

**Table 2:** Association between tremor severity and need for care.


MODEL STRUCTURE	TETRAS ADL SCORE		

	BIVARIATE^1^	CORE^2^	EXPANDED MODEL^3^

Logit model	1.0861***	1.0756***	1.0785***

	**TETRAS PERFORMANCE SCORE**		

	**BIVARIATE^1^**	**CORE^2^**	**EXPANDED MODEL^3^**

Logit model	1.0807***	1.0752***	1.0773***

	**TETRAS PERFORMANCE ITEM 4 SCORE**		

	**BIVARIATE^1^**	**CORE^2^**	**EXPANDED MODEL^3^**

Logit model	1.1899***	1.1760***	1.1801***


***p-value < 0.01; results presented in terms of odds ratios. ^1^Bivariate regression includes only TETRAS variable of interest with robust standard errors (Y = β_0_ + β_1_(TETRAS variable) + ɛ). ^2^Core model covariates also include age, sex, race, and CCI (Y = β_0_ + β_1_(TETRAS variable) + β_2_(AGE) + β_3_(SEX) + β_4_(RACE) + β_5_(CCI) + ɛ). ^3^Expanded model covariates also include core model covariates plus employment status (Y = β_0_ + β_1_(TETRAS variable) + β_2_(AGE) + β_3_(SEX) + β_4_(RACE) + β_5_(CCI) + β_6_(EMPLOYMENT_STATUS) + ɛ).

**Figure 4:**
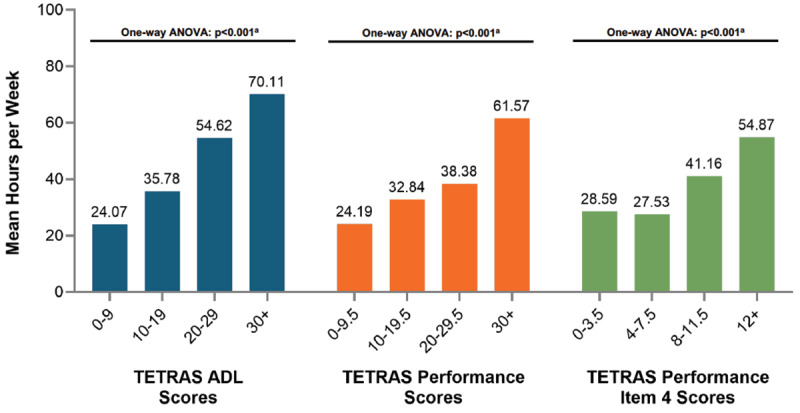
Association between caregiving time reported by care partners and tremor severity. ^a^One-way ANOVA testing a global null hypothesis that distributions are equal across the 4 subcategories within a given TETRAS measure. The p-values < 0.05 indicate that the mean hours per week is not equal across the subgroups.

From care partner/patient dyad data, the time required for providing care to ET patients increased with disease severity, as reflected by the increasing (i.e., worsening tremor) TETRAS-ADL, TETRAS-P, and TETRAS-P item 4 scores (One-way ANOVA, p < 0.001, Figure 4). Logistic regression models demonstrated a positive relationship between worsening TETRAS scores and the odds that the care partner provided 20 hours of care or more on average per week (Table 3), where age, sex, care partner’s relationship to patient, and care partner employment status were controlled for in the expanded model. Sensitivity analyses showed that the association remained intact for TETRAS-ADL and TETRAS-P when care partner age and care partner employment status were added to the models.

**Table 3:** Association between tremor severity and need for high-intensity caregiving (defined as greater than 20 hours per week).


MODEL STRUCTURE	TETRAS ADL SCORE		

	BIVARIATE^1^	CORE^2^	EXPANDED MODEL^3^

Logit model	1.0738***	1.0610***	1.0535***

	**TETRAS PERFORMANCE SCORE**		

	**BIVARIATE^1^**	**CORE^2^**	**EXPANDED MODEL^3^**

Logit model	1.0443***	1.0349**	1.0255*

	**TETRAS PERFORMANCE ITEM 4 SCORE**		

	**BIVARIATE^1^**	**CORE^2^**	**EXPANDED MODEL^3^**

Logit model	1.0996***	1.0670**	1.0471


***p-value < 0.01; ** p-value < 0.05; * p-value < 0.1; results presented in terms of odds ratios. ^1^Bivariate regression includes only TETRAS variable of interest with robust standard errors (Y = β_0_ + β_1_(TETRAS variable) + ɛ). ^2^Core model covariates also include age, sex, race, and CCI (Y = β_0_ + β_1_(TETRAS variable) + β_2_(AGE) + β_3_(SEX) + β_4_(RACE) + β_5_(CCI) + ɛ). ^3^Expanded model covariates also include core model covariates plus employment status (Y = β_0_ + β_1_(TETRAS variable) + β_2_(AGE) + β_3_(SEX) + β_4_(RACE) + β_5_(CCI) + β_6_(EMPLOYMENT_STATUS) + ɛ).

Additional resources were needed when caring for patients with ET. Forty-eight of the 239 care partners (20.1%) said they needed one or more additional resources, including a housekeeper or cleaner, shopping delivery services, gardening, and newspaper/magazine delivery. Furthermore, 83 of the 239 care partners (34.7%) reported one or more home modifications including bathroom adaptations, fitted grab bars or railing, kitchen adaptations, bedroom adaptations, and stair lift. Most (87.9%) low-intensity care partners providing less than 20 hours per week of caregiving felt additional help was not needed, while 64.9% of high-intensity care partners felt additional help was not needed.

## Discussion

Approximately 25% of patients in this clinic-based cohort required care partners, with average caregiving time ranging from 24 to 44 hours per week. Care partners were usually domestic partners or spouses. Caregiving intensity was not assessed with a standardized care partner burden instrument, but the number of hours reported by each care partner. In this study, 61.9% of care partners spent more than 20 hours per week providing care. Tremor severity, as assessed with TETRAS-P and TETRAS-ADL, was associated with increased odds of having a care partner and with increased hours spent on caregiving. These observations support a greater need for caregiving than has been previously reported in other cohorts.

Other studies have shown variable amounts of need for caregiving. Morgan et al. recruited 55 individuals with ET, ages 56-97, for a longitudinal study of cognitive function in ET and asked each participant to identify a care partner, defined as “a person who could provide perspective” on the participant’s well-being. However, 31% of these “care partners” reported providing no care, and only 11% reported more than 25 hours of care per week (mean 5.1 ± 9.9 SD) [13]. Care partner burden was positively correlated with cognitive impairment but not with tremor severity [13]. These findings may have been influenced by the objectives and inclusion criteria of the longitudinal study, which included ultimate brain donation, whereas our study recruited patients seeking care from their physicians in a clinic-based setting. Cognitive function was not assessed in our study.

In a German study comprising both an outpatient clinic sample and a community-based sample of individuals with ET, only 4% of individuals stated a “moderate to severe” need for help, though this was not quantified or defined in the study [24]. The authors reported that the questionnaire had not been validated and acknowledged that the interpretation of the results were limited by this [24].

In a study of 18 patients undergoing focused ultrasound thalamotomy, patients identified their primary care partner as “the individual providing the most assistance with tremor related challenges [25].” Thalamotomy reduced tremor significantly, and this was correlated with reduced need for assistance in ADLs [26]. However, the improvement in tremor was not correlated with perceived care partner burden measured with the 12-item Zarit Burden Interview Short Form (ZBI-12). The ZBI-12 was developed for the assessment of dementia caregiving and quantifies emotional, physical, and social impact on care partners. This scale was also used by Morgan et al. and may be suboptimal for assessing caregiving intensity in ET. Caregiving intensity was not measured as it was in the present study. Moreover, our analyses included only a small number of individuals receiving surgical therapies. Additional studies including patients receiving surgical therapies would be useful to clarify the relationship between surgical therapies, symptom relief, and caregiving intensity as well as care partner burden.

In our study and the aforementioned studies, most care partners were the patient’s spouse or domestic partner, with children and unrelated persons comprising the remainder [13]. On average, the care partners are younger and perceive greater patient suffering than the patients reported [13]. In general, care partner burden across various diseases is associated with report of need for greater help with daily tasks and may reflect the care partner’s ability to adapt to their new role [27]. The impact of these factors on the magnitude and prevalence of care partner burden could be considerable and merits further study in future ET treatment trials.

The generalizability of descriptive information from the DSP to all patients with ET may be limited [28]. Patients with more severe ET may make more frequent visits to their physician, and therefore, may be more likely to be included in the study population. Similarly, patients requiring greater care partner support are more likely to have their care partner with them to be recruited to this sample. In addition, we found that as expected, greater medical comorbidity has a highly significant influence on the need for care partner. However, these limitations should not invalidate our observation of a significant correlation between caregiving intensity and tremor severity. Furthermore, these data suggest that as individuals with ET experience worsening tremor severity, which may be mediated by a number of factors independent of medical co-morbidity and intrinsic to the experience of living with ET, including age at onset and longer disease duration [29,30,31,32,33], they may require care partners to help with activities of daily living as their disease progresses.

Our study had other limitations. Only one care partner perspective per patient was captured, but most care partners had one or more associate care partners. Although models adjusted for co-morbidity, it is not known precisely how much of the caregiving intensity was related specifically to ET. Reliance on an assumed number of hours for constant care (112 hours/week) may not accurately reflect the true effort provided by care partners but was deemed a reasonable estimate for the purpose of this study. Future studies could benefit from refined estimates of time spent caregiving, especially among those who live at home with the patient and whose primary role is caregiving.

In conclusion, many patients with ET require assistance from care partners in proportion to tremor severity. Over 50% of care partners in this study provided the equivalent of over half a work week. Unpaid work of this type has not been included in economic estimates of medical costs associated with ET [10,11,34] and merit further study.

## Additional File

The additional file for this article can be found as follows:

10.5334/tohm.1046.s1 Supplementary Table. Care partner demographics.

## References

[1] Louis ED, McCreary M. How Common is Essential Tremor? Update on the Worldwide Prevalence of Essential Tremor. Tremor and Other Hyperkinetic Movements. 2021 Jul 9;11(1):28. DOI: 10.5334/tohm.632 34277141 PMC8269764

[2] Bhatia KP, Bain P, Bajaj N, Elble RJ, Hallett M, Louis ED, et al. Consensus Statement on the classification of tremors. from the task force on tremor of the International Parkinson and Movement Disorder Society: IPMDS Task Force on Tremor Consensus Statement. Mov Disord. 2018 Jan;33(1):75–87. DOI: 10.1002/mds.27121 29193359 PMC6530552

[3] Louis ED, Gerbin M, Galecki M. Essential tremor 10, 20, 30, 40: clinical snapshots of the disease by decade of duration. Eur J Neurol. 2013 Jun;20(6):949–54. DOI: 10.1111/ene.12123 23521518 PMC3653981

[4] Louis ED, Benito-León J, Vega S, Bermejo-Pareja F, Group O, behalf of the ND in CS (NEDICES) S. Frailty in elderly persons with essential tremor: a population-based study (NEDICES). European Journal of Neurology. 2011;18(10):1251–7. DOI: 10.1111/j.1468-1331.2011.03374.x 21426443 PMC3135673

[5] Louis ED, Collins K, Rohl B, Morgan S, Robakis D, Huey ED, et al. Self-reported physical activity in essential tremor: Relationship with tremor, balance, and cognitive function. Journal of the Neurological Sciences. 2016 Jul 15;366:240–5. DOI: 10.1016/j.jns.2016.05.034 27288815 PMC4936779

[6] Gerbasi ME, Elble RJ, Jones E, Gillespie A, Jarvis J, Chertavian E, et al. Associations Among Tremor Amplitude, Activities of Daily Living, and Quality of Life in Patients with Essential Tremor. Tremor and Other Hyperkinetic Movements [Internet]. 2024 May 3 [cited 2024 Sep 25];14(1). Available from: https://tremorjournal.org/articles/10.5334/tohm.877 10.5334/tohm.877 PMC11067966 38708124

[7] Gültekin M, Biçer S, Çidem A, Baydemir R. Effects of Disability and Self-care Agency on the Activities of Daily Living in Patients with Essential Tremor. tnd. 2021 Dec 31;27(4):401–6. DOI: 10.4274/tnd.2021.44389

[8] Gerbasi ME, Nambiar S, Reed S, Hennegan K, Hadker N, Eldar-Lissai A, et al. Essential tremor patients experience significant burden beyond tremor: A systematic literature review. Front Neurol. 2022 Jul 22;13:891446. DOI: 10.3389/fneur.2022.891446 35937052 PMC9354397

[9] Huey ED, Cosentino S, Chapman S, Azar M, Rohl B, Collins K, et al. Self-report depressive symptoms are dissociated from tremor severity in essential tremor. Parkinsonism Relat Disord. 2018 May;50:87–93. DOI: 10.1016/j.parkreldis.2018.02.031 29499915 PMC5943134

[10] Kapinos KA, Louis ED. Annual health care costs among Medicare Beneficiaries with essential tremor. Parkinsonism & related disorders. 2022 Oct 3;104:26. DOI: 10.1016/j.parkreldis.2022.09.015 36206644 PMC9969906

[11] Dai D, Samiian A, Fernandes J, Coetzer H. Multiple Comorbidities, Psychiatric Disorders, Healthcare Resource Utilization and Costs Among Adults with Essential Tremor: A Retrospective Observational Study in a Large US Commercially Insured and Medicare Advantage Population. JHEOR. 2022 Aug 15;9(2):37–46. DOI: 10.36469/jheor.2022.37307 36051002 PMC9378814

[12] Kellner S, Morgan S, Gutierrez J, Collins K, Rohl B, Migliore F, et al. Perceived Embarrassment and Caregiver Burden in Essential Tremor Caregivers. J Neurol Sci. 2017 Dec 15;383:205–10. DOI: 10.1016/j.jns.2017.11.020 29246614 PMC5739080

[13] Morgan S, Kellner S, Gutierrez J, Collins K, Rohl B, Migliore F, et al. The Experience of Essential Tremor Caregivers: Burden and Its Correlates. Front Neurol. 2017;8:396. DOI: 10.3389/fneur.2017.00396 28855888 PMC5557742

[14] Cersonsky TEK, Diaz DT, Kellner S, Hickman R, Zdrodowska MA, Monin JK, et al. Enfeeblement in Elders with Essential Tremor: Characterizing the Phenomenon and Its Role in Caregiver Burden. Tremor Other Hyperkinet Mov (N Y). 2019 Oct 18;9. DOI: 10.5334/tohm.461 PMC6814912 31709127

[15] Liu Z, Heffernan C, Tan J. Caregiver burden: A concept analysis. Int J Nurs Sci. 2020 Jul 25;7(4):438–45. DOI: 10.1016/j.ijnss.2020.07.012 33195757 PMC7644552

[16] Xu L, Liu Y, He H, Fields NL, Ivey DL, Kan C. Caregiving intensity and caregiver burden among caregivers of people with dementia: The moderating roles of social support. Archives of Gerontology and Geriatrics. 2021 May 1;94:104334. DOI: 10.1016/j.archger.2020.104334 33516077

[17] Anderson P, Benford M, Harris N, Karavali M, Piercy J. Real-world physician and patient behaviour across countries: Disease-Specific Programmes – a means to understand. Curr Med Res Opin. 2008 Nov;24(11):3063–72. DOI: 10.1185/03007990802457040 18826746

[18] Kumagai N. Distinct impacts of high intensity caregiving on caregivers’ mental health and continuation of caregiving. Health Economics Review. 2017 Apr 7;7(1):15. DOI: 10.1186/s13561-017-0151-9 28389976 PMC5383799

[19] Zhang Y, Bennett MR. Insights Into Informal Caregivers’ Well-being: A Longitudinal Analysis of Care Intensity, Care Location, and Care Relationship. The Journals of Gerontology: Series B. 2024 Feb 1;79(2):gbad166. DOI: 10.1093/geronb/gbad166 PMC10832593 38299971

[20] Jawahir S, Tan EH, Tan YR, Mohd Noh SN, Ab Rahim I. The impacts of caregiving intensity on informal caregivers in Malaysia: findings from a national survey. BMC Health Services Research. 2021 Apr 27;21(1):391. DOI: 10.1186/s12913-021-06412-5 33906646 PMC8077883

[21] Ramsay S, Grundy E, O’Reilly D. The relationship between informal caregiving and mortality: an analysis using the ONS Longitudinal Study of England and Wales. J Epidemiol Community Health. 2013 Aug;67(8):655–60. DOI: 10.1136/jech-2012-202237 23737544

[22] Elble RJ. The Essential Tremor Rating Assessment Scale. Journal of Neurology. 2016;5.

[23] Quan H, Li B, Couris CM, Fushimi K, Graham P, Hider P, et al. Updating and validating the Charlson comorbidity index and score for risk adjustment in hospital discharge abstracts using data from 6 countries. Am J Epidemiol. 2011 Mar 15;173(6):676–82. DOI: 10.1093/aje/kwq433 21330339

[24] Lorenz D, Poremba C, Papengut F, Schreiber S, Deuschl G. The psychosocial burden of essential tremor in an outpatient- and a community-based cohort: The Psychosocial Burden of Essential Tremor. European Journal of Neurology. 2011 Jul;18(7):972–9. DOI: 10.1111/j.1468-1331.2010.03295.x 21244579

[25] Gopinath G, Scantlebury N, Sewell IJ, Rohringer CR, Sivadas S, McSweeney M, et al. Changes in Caregiver Burden Following Unilateral Magnetic Resonance-Guided Focused Ultrasound Thalamotomy for Essential Tremor. Mov Disord Clin Pract. 2024 Apr 4;11(7):905–8. DOI: 10.1002/mdc3.14034 38576099 PMC11233863

[26] Bédard M, Molloy DW, Squire L, Dubois S, Lever JA, O’Donnell M. The Zarit Burden Interview: a new short version and screening version. Gerontologist. 2001 Oct;41(5):652–7. DOI: 10.1093/geront/41.5.652 11574710

[27] Garlo K, O’Leary JR, Van Ness PH, Fried TR. Burden in Caregivers of Older Adults with Advanced Illness. J American Geriatrics Society. 2010 Dec;58(12):2315–22. DOI: 10.1111/j.1532-5415.2010.03177.x PMC3058825 21087225

[28] Babineaux SM, Curtis B, Holbrook T, Milligan G, Piercy J. Evidence for validity of a national physician and patient-reported, cross-sectional survey in China and UK: the Disease Specific Programme. BMJ Open. 2016 Aug;6(8):e010352. DOI: 10.1136/bmjopen-2015-010352 PMC5013497 27531722

[29] Hopfner F, Ahlf A, Lorenz D, Klebe S, Zeuner KE, Kuhlenbäumer G, et al. Early- and late-onset essential tremor patients represent clinically distinct subgroups. Mov Disord. 2016 Oct;31(10):1560–6. DOI: 10.1002/mds.26708 27384030

[30] Putzke JD, Whaley NR, Baba Y, Wszolek ZK, Uitti RJ. Essential tremor: predictors of disease progression in a clinical cohort. J Neurol Neurosurg Psychiatry. 2006 Nov;77(11):1235–7. DOI: 10.1136/jnnp.2006.086579 17043291 PMC2077383

[31] Gironell A, Ribosa-Nogué R, Gich I, Marin-Lahoz J, Pascual-Sedano B. Severity Stages in Essential Tremor: A Long-Term Retrospective Study Using the Glass Scale. Tremor Other Hyperkinet Mov (N Y). 2015 Mar 13;5:299. DOI: 10.5334/tohm.245 25793146 PMC4361372

[32] Louis ED, Faust PL, Vonsattel JPG, Honig LS, Henchcliffe C, Pahwa R, et al. Older Onset Essential Tremor: More Rapid Progression and More Degenerative Pathology. Mov Disord. 2009 Aug 15;24(11):1606–12. DOI: 10.1002/mds.22570 19526587 PMC2736358

[33] Louis ED, Hernandez N, Ionita-Laza I, Ottman R, Clark LN. Does Rate of Progression Run in Essential Tremor Families? Slower vs. Faster Progressors. Parkinsonism Relat Disord. 2013 Mar;19(3):363–6. DOI: 10.1016/j.parkreldis.2012.10.005 23121728 PMC3578031

[34] Kapinos KA, Louis ED. The Direct Medical Cost of Essential Tremor. Neuroepidemiology. 2024 Oct 11;1–7. DOI: 10.1159/000541968 39396507

