# Supplementary material for: Association Between Tremor Severity and Caregiving Intensity in Essential Tremor
Source: Tremor Other Hyperkinet Mov (N Y). 2025 Aug 7;15:37. doi: 10.5334/tohm.1046 (PMC12330806; doi:10.5334/tohm.1046)
Supplement: Supplementary Table. — Care partner demographics. [file tohm-15-1-1046-s1.pdf]

Supplementary Table. Care partner demographics.

		Patient/Care Partner cohort (N = 239)	
Care partner Age (years), mean (sd)	Care partner Age	56.9	(14.4)
High caregiving intensity (%)[1]	Yes	61.9%	(148)
Caregiving hours per week, mean (sd)[2]	Hours per week	24.5	(26.1)
Care partner employment status (%)	Full time	38.9%	(93)
	Part time/student	22.2%	(53)
	Retired	23.4%	(56)
	Unemployed/NS	15.5%	(37)
Care partner marital status (%)	Married	74.1%	(177)
	Divorced/separated	8.8%	(21)
	Widowed	5.0%	(12)
	Single/never married	10.0%	(24)
	Other	1.7%	(4)
	None selected	0.4%	(1)
Relationship to patient (%)[3]	Partner/spouse	61.1%	(146)
	Professional	8.4%	(20)
	Child	16.3%	(39)
	Other family	5.4%	(13)
	Other	8.8%	(21)
Caretaker lives with patient? (%)	Yes	73.2%	(175)
	Yes, some of the time	8.8%	(21)
	No	15.9%	(38)
	None selected	2.1%	(5)

Number of other care partners (%)	0	7.5%	(18)
	1	81.2%	(194)
	2	10.0%	(24)
	3	1.3%	(3)
Other professional care partner (%)[4]		5.9%	(14)
Other live-in care partner (%)[5]		13.4%	(32)

[1]A care partner was categorized as having a high burden if 1) he/she reported giving "constant" care or 2) he/she provided at least 20 hours of caregiving per week

[2]Caregiving hours per week for care partners reporting either average weekly hours or average daily hours. Care partners giving "constant" care were not included.

[3]Professional = nursing home staff, nurse, home help, or professional care partner; Other = voluntary care partner, friend/neighbor, other

[4]Nurse, home help, or professional care partner

[5]Nursing home staff or spouse/partner
